# Supplementary material for: Lipoprotein Deprivation Reveals a Cholesterol-Dependent Therapeutic Vulnerability in Diffuse Glioma Metabolism
Source: Cancers (Basel). 2022 Aug 11;14(16):3873. doi: 10.3390/cancers14163873 (PMC9405833; doi:10.3390/cancers14163873)
Supplement: Supplementary file 1 [file cancers-14-03873-s001.zip › cancers-1834600-supplementary.pdf]

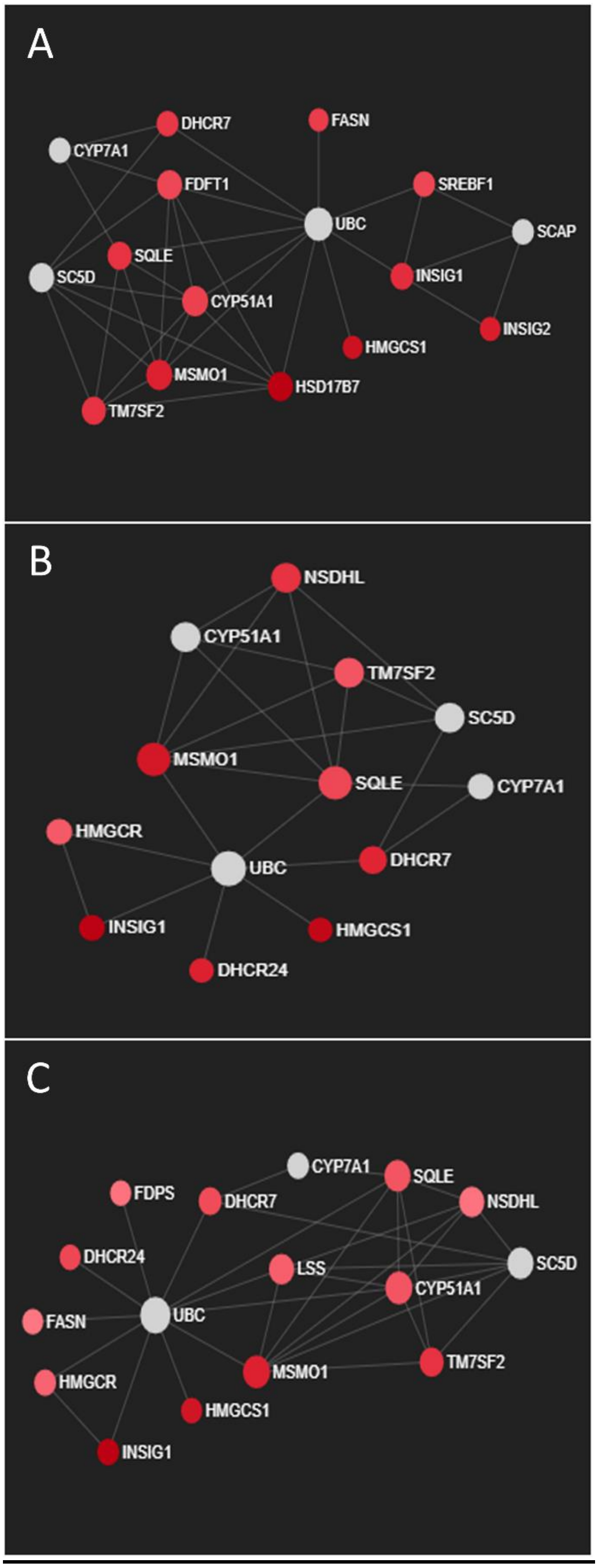

Supplementary Figure S1. Cholesterol-related transcriptomic networks induced by lipoprotein deprivation. Extent of upregulated gene expression in U373 (A), KNS42 (B), and

SF188 (C) cells represented by red colour intensity. Grey nodes represent non-significant genes required to construct the minimum network connecting all indicated significant genes.

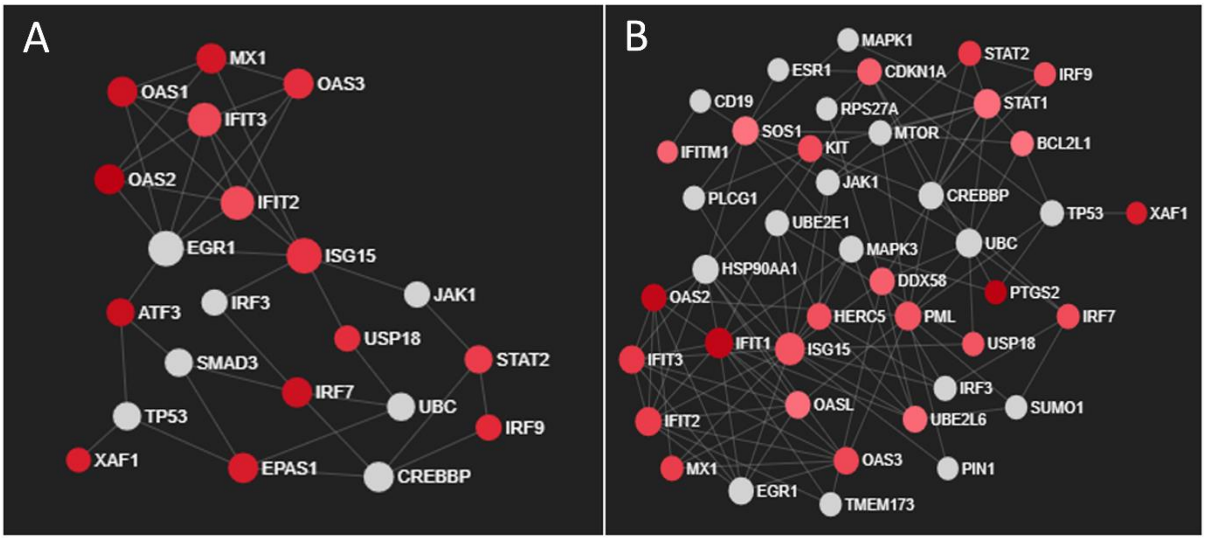

Supplementary Figure S2. Interferon-related transcriptomic networks induced by lipoprotein deprivation. Extent of upregulated gene expression in KNS42 (A) and SF188 (B) cells represented by red colour intensity. Grey nodes represent non-significant genes required to construct the minimum network connecting all indicated significant genes.

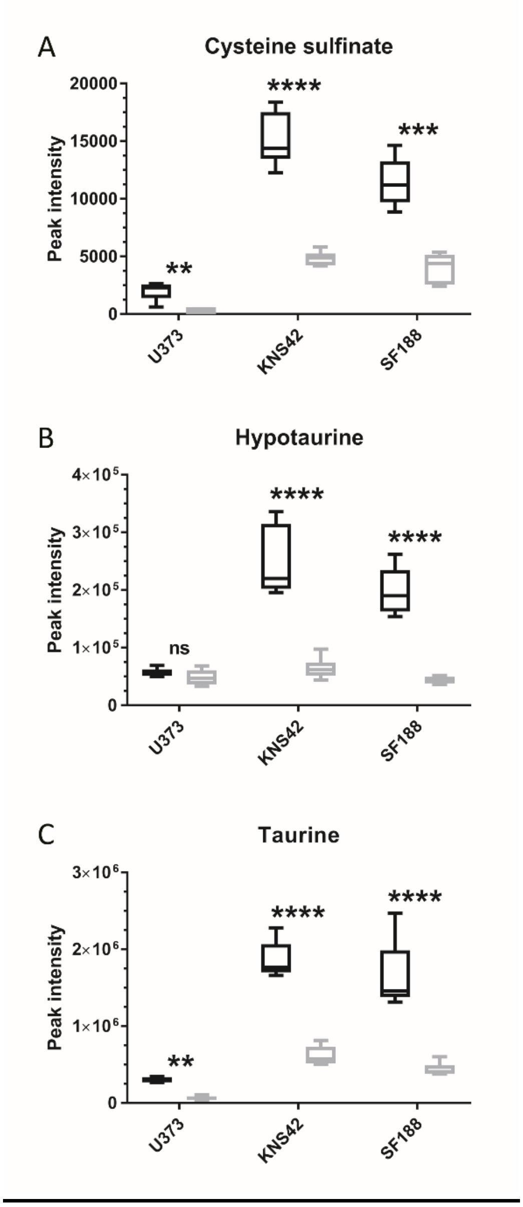

Supplementary Figure S3. Peak intensities of cysteine sulfinate, hypotaurine, and taurine in diffuse glioma cells starved of lipoproteins. Mean peak intensities are displayed for taurine-related metabolites under lipoprotein-replete (NM; red) or -deplete (LPDM; green) conditions in U373, KNS42, and SF188 cells. Results are the mean ± SD of n=6 independent replicates. Statistical evaluation was performed using a *t*-test: ns = not significant; * $p<0.05$; ** $p<0.01$; *** $p<0.001$; **** $p<0.0001$.

Supplementary Table S1. Peak intensity fold changes for metabolites within the glycolysis pathway, tricarboxylic acid cycle, and amino acid metabolism under lipoprotein-replete relative to lipoprotein-deplete conditions.

Metabolite	ID confidence	U373		KNS42		SF188	
		log2FC	FDR	log2FC	FDR	log2FC	FDR
Glycolysis							
D-Glucose 6-phosphate	10	-0.04	9.20E-01	0.01	9.99E-01	-0.24	4.83E-01
D-Glyceraldehyde 3-phosphate	8	2.45	**2.12E-05**	0.06	9.95E-01	-1.60	5.22E-02
3-Phospho-D-glycerate	8	-0.92	8.53E-02	-0.12	8.93E-01	-0.01	4.94E-01
Phosphoenolpyruvate	8	-1.53	**2.06E-02**	-0.09	9.97E-01	0.90	2.16E-01
Pyruvate	8	-0.36	3.28E-01	-1.20	**2.00E-02**	-1.66	**5.10E-03**
Lactate	10	0.70	7.73E-02	0.01	9.91E-01	-0.15	6.45E-01
Tricarboxylic acid cycle							
Citrate	8	0.50	8.52E-02	0.35	**3.62E-02**	0.38	5.82E-02
Succinate	10	-1.63	**6.11E-05**	-0.79	**1.58E-02**	0.20	1.11E-01
Malate	10	-0.10	6.78E-01	-0.34	**2.56E-02**	-0.66	**2.43E-04**
Energetics							
ADP	10	0.21	7.36E-01	0.24	7.33E-01	0.12	7.58E-01
ATP	8	0.80	**4.17E-02**	-0.05	9.72E-01	0.38	4.14E-01
NAD+	8	0.57	3.22E-01	0.17	6.44E-01	0.28	1.92E-01
NADH	8	-0.04	9.98E-01	-0.28	2.84E-01	-0.28	5.32E-02
NADPH	8	0.21	7.25E-01	-0.05	8.45E-01	-0.30	5.29E-01
Essential amino acids							
L-Histidine	10	0.11	4.60E-01	0.28	5.01E-02	0.44	**2.72E-03**
L-Isoleucine	10	0.13	1.15E-01	0.11	2.56E-01	0.31	**1.89E-03**
L-Lysine	8	0.27	4.42E-01	0.14	8.04E-01	0.23	5.22E-01
L-Methionine	10	0.08	1.88E-01	0.13	1.25E-01	0.31	**8.67E-05**
L-Threonine	10	0.21	**3.47E-02**	0.28	**5.71E-04**	0.24	**1.14E-03**
L-Tryptophan	10	0.16	2.03E-01	0.23	**1.20E-02**	0.36	**2.13E-04**
L-Valine	8	0.06	5.87E-01	0.18	7.20E-02	0.33	**9.60E-04**
Conditional amino acids							
L-Arginine	10	-0.03	9.20E-01	-0.09	6.40E-01	0.18	3.47E-01
L-Cysteine	8	-0.16	8.82E-01	0.70	1.67E-01	0.42	3.37E-01
L-Glutamine	10	-0.07	7.31E-01	0.41	**6.39E-03**	0.38	**7.99E-03**
L-Tyrosine	10	0.28	**3.15E-02**	0.45	**1.00E-03**	0.62	**1.33E-04**
Glycine	10	0.40	7.84E-02	0.36	9.47E-02	0.84	**9.84E-04**
L-Proline	10	0.29	**2.89E-02**	0.21	6.99E-02	0.48	**7.22E-04**
L-Serine	10	-0.54	**1.12E-02**	-0.53	**3.48E-04**	-0.55	**1.46E-03**
Non-essential amino acids							
L-Alanine	8	-0.37	9.35E-02	-0.73	**4.16E-04**	-0.76	**2.10E-05**
L-Asparagine	10	-0.14	1.65E-01	0.42	**6.84E-03**	0.72	**4.14E-04**
L-Aspartate	10	-0.12	3.44E-01	0.25	1.65E-01	0.22	5.32E-02
L-Glutamate	10	0.21	8.98E-02	0.19	1.64E-01	0.33	**8.38E-04**

Fold change differences are given to the base of 2 (log2FC). Statistical evaluation was performed using *t*-test and *p*-values were corrected for multiple comparisons using the false discovery rate (FDR). Significant scores are highlighted in bold.

Supplementary Table S2. Peak intensity fold changes for metabolites within the methionine cycle, proline synthesis pathway, and choline metabolism under lipoprotein-replete relative to lipoprotein-deplete conditions.

Metabolite	ID confidence	U373		KNS42		SF188	
		log2FC	FDR	log2FC	FDR	log2FC	FDR
Methionine cycle/transulfuration pathway							
L-Methionine	10	0.08	1.88E-01	0.13	1.25E-01	0.31	**5.60E-02**
S-Adenosyl-L-methionine	8	0.43	7.05E-01	0.10	9.42E-01	-0.19	8.94E-01
S-Adenosyl-L-homocysteine	8	-0.14	9.02E-01	-0.09	8.41E-01	0.39	4.35E-01
L-Serine	10	-0.54	**1.12E-02**	-0.53	**3.48E-04**	-0.55	**1.46E-03**
L-Cystathionine	10	-1.02	**9.66E-03**	-0.78	**5.32E-04**	-0.92	**7.75E-03**
L-Cysteine	8	-0.16	8.82E-01	0.70	1.67E-01	0.42	3.37E-01
Glycine	10	0.40	7.84E-02	0.36	9.47E-02	0.84	**9.84E-04**
γ-L-Glutamyl-L-cysteine	8	0.10	9.64E-01	0.11	9.85E-01	0.29	6.97E-01
Glutathione	8	0.16	9.44E-01	-0.35	4.40E-01	-0.74	5.98E-02
Glutathione disulfide	10	0.55	2.25E-01	0.14	6.09E-01	0.13	7.75E-01
L-Cysteine sulfinate	7	-2.69	**1.30E-03**	-1.64	**2.46E-06**	-1.50	**8.68E-04**
Hypotaurine	8	-0.22	3.28E-01	-1.94	**5.19E-05**	-2.18	**2.32E-06**
Taurine	10	-2.16	**1.16E-03**	-1.60	**2.61E-05**	-1.92	**1.57E-05**
Proline metabolism pathway							
Glutamine	10	-0.07	7.31E-01	0.41	**6.39E-03**	0.38	**7.99E-03**
L-Glutamate	10	0.21	8.98E-02	0.19	1.64E-01	0.33	**8.38E-04**
L-Glutamate 5-semialdehyde	8	-3.14	**6.11E-05**	-1.94	**8.49E-05**	-2.22	**1.17E-07**
1-Pyrroline-5-carboxylate	8	0.18	3.21E-01	0.33	**2.79E-02**	0.58	**1.43E-04**
L-Proline	10	0.29	**2.89E-02**	0.21	6.99E-02	0.48	**7.22E-04**
Choline metabolism pathway							
Glycerophosphocholine	10	-0.11	2.95E-01	-0.34	**3.70E-02**	-0.56	**9.41E-04**
Choline	8	-0.99	**1.59E-02**	-1.33	**4.13E-03**	-1.34	**5.95E-06**
Choline phosphate	10	-1.09	**1.11E-03**	0.00	9.91E-01	-0.03	9.10E-01
CDP-choline	8	0.80	3.34E-01	0.94	**2.56E-02**	1.47	**3.32E-04**

Fold change differences are given to the base of 2 (log2FC). Statistical evaluation was performed using *t*-test and *p*-values were corrected for multiple comparisons using the false discovery rate (FDR). Significant scores are highlighted in bold.

Supplementary Table S3. Peak intensity fold changes for metabolites within the methionine cycle, proline synthesis pathway, and choline metabolism under lipoprotein-replete relative to lipoprotein-deplete conditions.

Metabolite	ID confidence	U373		KNS42		SF188	
		log2FC	FDR	log2FC	FDR	log2FC	FDR
Building blocks							
Ribose 5-phosphate	10	0.56	7.93E-01	0.66	9.51E-02	1.10	**7.06E-03**
Glutamine	10	-0.07	7.31E-01	0.41	**6.39E-03**	0.38	**7.99E-03**
Glycine	10	0.40	7.84E-02	0.36	9.47E-02	0.84	**9.84E-04**
Aspartate	10	-0.12	3.44E-01	0.25	1.65E-01	0.22	5.32E-02
Purine synthesis							
FGAR	8	-0.54	1.21E-01	-1.72	**1.96E-02**	-3.18	**6.89E-06**
IMP	10	0.33	5.38E-01	-0.20	6.49E-01	-0.65	5.48E-02
GMP	10	0.47	4.67E-01	-0.11	7.98E-01	-0.53	1.91E-01
AMP	10	0.07	8.82E-01	-0.49	1.61E-01	-0.92	**2.92E-03**
ADP	10	0.21	7.36E-01	0.24	7.33E-01	0.12	7.58E-01
ATP	8	0.80	**4.17E-02**	-0.05	9.72E-01	0.38	4.14E-01
Purine degradation							
Adenosine	10	-0.77	**3.40E-02**	-0.72	9.47E-02	-0.90	**3.07E-02**
Guanosine	10	-0.81	1.68E-01	-0.55	5.09E-01	-0.37	4.96E-01
Hypoxanthine	10	-0.60	**3.99E-02**	-0.20	7.74E-01	0.47	5.72E-01
Xanthine	10	2.34	**4.98E-03**	3.35	**7.39E-05**	4.42	**9.42E-06**
Uric acid	10	-4.65	**7.63E-05**	-3.09	**5.94E-05**	-3.50	**1.05E-05**
Pyrimidine synthesis							
Carbamoylaspartate	8	0.03	9.98E-01	-0.82	**2.07E-02**	-1.83	**1.26E-04**
Dihydroorotate	8	-0.04	9.04E-01	-1.00	**7.60E-03**	-2.09	**1.43E-04**
Orotate	10	-0.75	6.14E-02	-0.90	**2.31E-02**	-1.94	**5.84E-04**
UMP	10	0.31	7.02E-01	0.52	6.58E-01	0.48	6.28E-01
UDP	10	-0.35	8.90E-01	0.45	4.67E-01	0.42	1.34E-01
Salvage pathways							
Hypoxanthine	10	-0.60	**3.99E-02**	-0.20	7.74E-01	0.47	5.72E-01
Adenine	10	0.22	6.85E-01	-0.22	5.66E-01	-0.38	7.37E-02
Cytidine	8	0.34	3.21E-01	-0.19	5.69E-01	0.29	5.41E-01
Thymine	10	-0.04	8.35E-01	0.54	1.97E-01	1.08	**4.85E-04**
Uridine	8	-0.25	5.71E-01	-0.13	6.27E-01	0.16	6.12E-01

Fold change differences are given to the base of 2 (log2FC). Statistical evaluation was performed using *t*-test and *p*-values were corrected for multiple comparisons using the false discovery rate (FDR). Significant scores are highlighted in bold.

Supplementary Table S4. Gene ontology analysis of differentially expressed genes in LXR-623 treated U373 cells.

GO.ID	Term	Annotated	Significant	Expected	classicFisher	weight01KS	Genes
GO:0006915	apoptotic process	1100	103	49.63	8.80E-14	8.80E-03	NOG , VEGFA , GDF15 , CSF2 , **ID3** , ADM , CHAC1 , **EDNRB** , NDRG1 , ASNS , DDIT3 , **TAF9B** , **ID1** , MT3 , PPP1R15A , **CNR1** , PHLDA2 , IER3 , **PDGFRB** , TBX3 , F3 , IRS2 , ICAM1 , CTH , USP53 , MKNK2 , MYC , KITLG , FAM162A , TRIB3
GO:0042127	regulation of cell proliferation	903	92	40.74	1.80E-14	1.93E-02	NOG , VEGFA , CSF2 , CXCL8 , ADM , **EDNRB** , **PDGFRA** , NDRG1 , LIF , **ID1** , E2F7 , PHLDA2 , **PDGFRB** , TBX3 , MXI1 , F3 , IRS2 , ADAMTS1 , MYC , KITLG , NAMPT , **TXNIP** , ARHGEF2 , CEBPB , SPRY2 , HMOX1 , **S100B** , **ASPM** , **PDE5A**
GO:0008283	cell proliferation	1124	107	50.71	7.70E-15	2.02E-02	NOG , VEGFA , CSF2 , CXCL8 , ADM , **EDNRB** , **PDGFRA** , NDRG1 , LIF , **LGR4** , **ID1** , **NDP** , MT3 , E2F7 , PHLDA2 , **PDGFRB** , TBX3 , MXI1 , F3 , IRS2 , ADAMTS1 , MYC , KITLG , NAMPT , **TXNIP** , ARHGEF2 , CEBPB , SPRY2 , HMOX1 , **S100B**
GO:0042325	regulation of phosphorylation	843	85	38.03	4.20E-13	3.93E-02	NOG , VEGFA , GDF15 , CSF2 , **EDNRB** , **PDGFRA** , LIF , **ID1** , MT3 , DUSP5 , **PIK3R3** , PPP1R15A , SESN2 , SRPX2 , **PDGFRB** , SPINK1 , IRS2 , SEMA7A , ICAM1 , MYC , KITLG , TRIB3 , ARHGEF2 , SPRY2 , GPRC5A , **PDE5A** , DDIT4 , TNFRSF10B , **CENPE**, SPRY4
GO:0035556	intracellular signal transduction	1501	133	67.72	3.90E-16	1.53E-01	ABCA1 , VEGFA , GDF15 , CSF2 , CXCL8 , ADM , CHAC1 , **EDNRB** , **PDGFRA** , NDRG1 , **SESN3** , LIF , RND3 , DDIT3 , **TAF9B** , **ID1** , **LPAR4** , EIF4EBP1 , MT3 , E2F7 , DUSP5 , PPP1R15A , SESN2 , IER3 , **PDGFRB** , **SPAAR** , SPINK1 , F3 , IRS2 , SEMA7A

Statistical evaluation of gene ontology (GO) categories based of the number of significant-to-expected observations using either Fisher's exact test (Fis) or Kolmogorov-Smirnov test (KS). Classic and weight01 algorithms were utilised which do not or do take into account of the structure of the GO hierarchy, respectively. Genes in bold were downregulated under lipoprotein-deplete conditions.

Supplementary Table S5. Gene ontology analysis of differentially expressed genes in LXR-623 treated KNS42 cells.

GO.ID	Term	Annotated	Significant	Expected	classicFisher	weight01KS	Genes
GO:0007062	sister chromatid cohesion	113	65	23.04	3.70E-18	1.70E-16	NDC80 , CENPI , CENPE , SKA2 , TNKS , ZWILCH , BIRC5 , SPC24 , KNTC1 , FEN1 , KIF18A , KNL1 , AURKB , CENPK , CENPU , BUB1 , CENPL , SPC25 , MAD2L1 , MIS12 , DSN1 , SGO1 , CDCA8 , CHTF8 , CENPM , POGZ , CENPC , SLF1 , CENPF , DSCC1
GO:0051301	cell division	434	165	88.51	4.50E-18	1.90E-15	HMCN1 , CCNE2 , NDC80 , KIF11 , CENPE , CCNA2 , BRIP1 , ASPM , NUSAP1 , NCAPG , HELLS , BRCA2 , KIF20A , CDC6 , OIP5 , SKA2 , ANLN , SMC2 , TNKS , ZWILCH , SKA3 , BIRC5 , SPAG5 , SPC24 , KNTC1 , KIF4A , TPX2 , TOP2A , CCNB1 , CDCA2
GO:0006260	DNA replication	239	101	48.74	6.80E-15	5.30E-05	CLSPN , CCNE2 , GINS2 , RFWD3 , BMP4 , POLQ , RMI1 , BRIP1 , DTL , TICRR , BRCA2 , CDC6 , ORC1 , PCLAF , MCM8 , GMNN , RRM2 , MCM10 , GINS1 , CHAF1B , CHEK1 , EXO1 , POLA2 , DNA2 , FEN1 , RRM1 , PCNA , ESCO2 , CDC7 , POLA1
GO:0007049	cell cycle	1372	450	279.8	< 1e-30	0.00025	SKP2 , HMCN1 , CLSPN , DLGAP5 , CCNE2 , GINS2 , NDC80 , KIF11 , STIL , CENPI , RFWD3 , DIRAS3 , BMP4 , RBL1 , CENPE , MKI67 , WDR76 , CCNA2 , PDGFRB , BRIP1 , ASPM , NUSAP1 , HMMR , NCAPG , DTL , FANCI , TICRR , HELLS , BRCA2 , MYBL2
GO:0000070	mitotic sister chromatid segregation	120	62	24.47	2.20E-14	0.00055	DLGAP5 , NDC80 , CENPE , NUSAP1 , NCAPG , CDC6 , SMC2 , TNKS , SPAG5 , KIF4A , KIF18A , CCNB1 , AURKB , NCAPD3 , BUB1 , MAD2L1 , MIS12 , GEN1 , DSN1 , SGO1 , KIF14 , KIF18B , CDCA8 , CHTF8 , POGZ , KIFC1 , NCAPH , CENPC , SLF1 , CDC23

Statistical evaluation of gene ontology (GO) categories based of the number of significant-to-expected observations using either Fisher's exact test (Fis) or Kolmogorov-Smirnov test (KS). Classic and weight01 algorithms were utilised which do not or do take into account of the structure of the GO hierarchy, respectively. Genes in bold were downregulated under lipoprotein-deplete conditions.

Supplementary Table S6. Gene ontology analysis of differentially expressed genes in LXR-623 treated SF188 cells.

GO.ID	Term	Annotated	Significant	Expected	classicFisher	weight01KS	Genes
GO:0051301	cell division	368	154	83.85	7.20E-17	1.80E-09	***CCNF*** , ***SPC25*** , ***KIF20A*** , ***HELLS*** , *VEGFA* , ***CCNA2*** , ***KIF11*** , ***CCNE2*** , ***NCAPG*** , ***FAM83D*** , ***BRIP1*** , ***SKA3*** , ***BUB1*** , *EREG* , ***NUSAP1*** , ***CENPE*** , ***ASPM*** , ***PLK1*** , ***ANLN*** , ***CDCA2*** , ***NDC80*** , ***ESPL1*** , ***OIP5*** , ***NCAPH*** , ***SPC24*** , ***CDK2*** , ***CCNB1*** , ***SKA2*** , ***TOP2A*** , ***BRCC3*** , ***CDC7***
GO:0006260	DNA replication	206	105	46.94	4.30E-19	4.70E-05	***PCLAF*** , ***ESCO2*** , ***CHAF1B*** , ***CCNE2*** , ***BMP4*** , ***CLSPN*** , ***MCM10*** , ***BRIP1*** , *EREG* , ***DTL*** , ***GINS3*** , ***GINS2*** , ***RMI1*** , ***MCM5*** , ***EXO1*** , ***RRM2*** , ***GINS1*** , ***CDK2*** , ***TICRR*** , ***MCM3*** , ***PCNA*** , ***RFC5*** , ***CDC7*** , ***CDC25C*** , ***CCNE1*** , ***E2F8*** , ***DNA2*** , ***GINS4*** , ***CDC25A*** , ***NASP***
GO:0000278	mitotic cell cycle	685	275	156.08	2.50E-26	7.30E-05	***SKP2*** , ***MKI67*** , ***SPC25*** , *ASNS* , ***KIF20A*** , ***EDN1*** , ***DLGAP5*** , ***CCNA2*** , ***KIF11*** , ***CCNE2*** , ***NCAPG*** , ***PDGFRB*** , *CDKN2B* , ***BMP4*** , ***CLSPN*** , ***MCM10*** , ***GTSE1*** , ***SKA3*** , ***BUB1*** , *EREG* , ***NUSAP1*** , ***CENPE*** , ***PLK1*** , ***ANLN*** , ***DTL*** , ***GINS2*** , ***NDC80*** , ***MCM5*** , ***MYBL2*** , ***ESPL1***
GO:0007049	cell cycle	1186	439	270.24	< 1e-30	0.00013	***SKP2*** , *DDIT3* , *NUPR1* , ***MKI67*** , ***PCLAF*** , ***CCNF*** , ***SPC25*** , *ASNS* , ***KIF20A*** , *PTGS2* , ***EDN1*** , ***CENPI*** , ***DLGAP5*** , ***ESCO2*** , ***HELLS*** , ***CHAF1B*** , ***WDR76*** , ***CCNA2*** , ***KIF11*** , ***CCNE2*** , ***NCAPG*** , ***FAM83D*** , ***PDGFRB*** , *CDKN2B* , ***BMP4*** , ***CLSPN*** , ***MCM10*** , ***BRIP1*** , ***GTSE1*** , ***SKA3***
GO:0051726	regulation of cell cycle	762	261	173.63	3.10E-14	0.0013	***SKP2*** , *DDIT3* , *NUPR1* , ***MKI67*** , ***PCLAF*** , ***CCNF*** , *ASNS* , *PTGS2* , ***EDN1*** , ***DLGAP5*** , ***WDR76*** , ***CCNA2*** , ***KIF11*** , ***CCNE2*** , ***FAM83D*** , ***PDGFRB*** , *CDKN2B* , ***BMP4*** , ***CLSPN*** , ***BRIP1*** , ***GTSE1*** , ***BUB1*** , *EREG* , ***NUSAP1*** , ***CENPE*** , ***ASPM*** , ***PLK1*** , ***ANLN*** , *LIF* , ***DTL***

Statistical evaluation of gene ontology (GO) categories based of the number of significant-to-expected observations using either Fisher's exact test (Fis) or Kolmogorov-Smirnov test (KS). Classic and weight01 algorithms were utilised which do not or do take into account of the structure of the GO hierarchy, respectively. Genes in bold were downregulated under lipoprotein-deplete conditions.
