# Supplementary figures and images for: Lipoprotein Deprivation Reveals a Cholesterol-Dependent Therapeutic Vulnerability in Diffuse Glioma Metabolism
Source: Cancers (Basel). 2022 Aug 11;14(16):3873. doi: 10.3390/cancers14163873 (PMC9405833; doi:10.3390/cancers14163873)

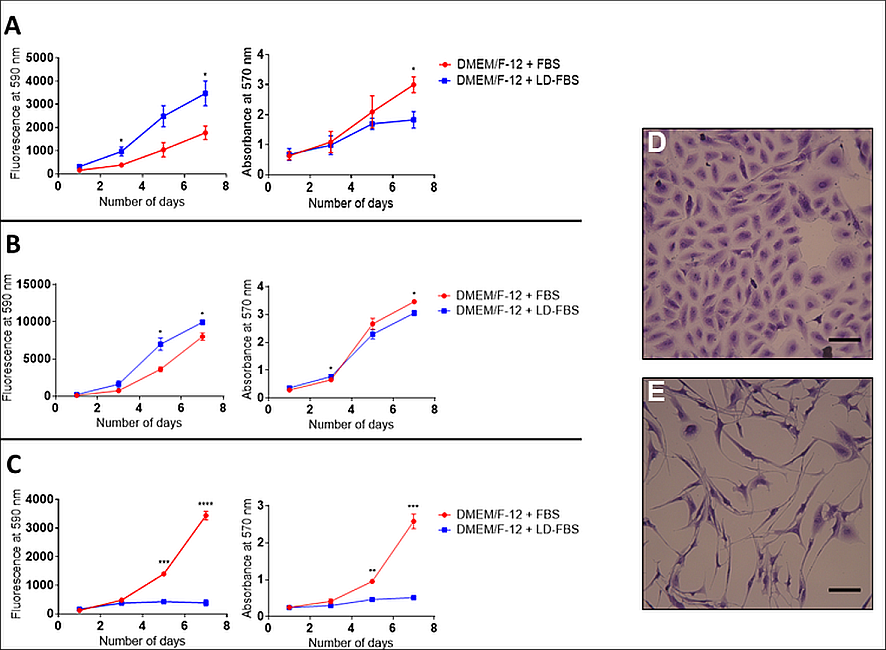

Supplement: Supplementary file 1 [file cancers-14-03873-s001.zip › Figure S1.tif]

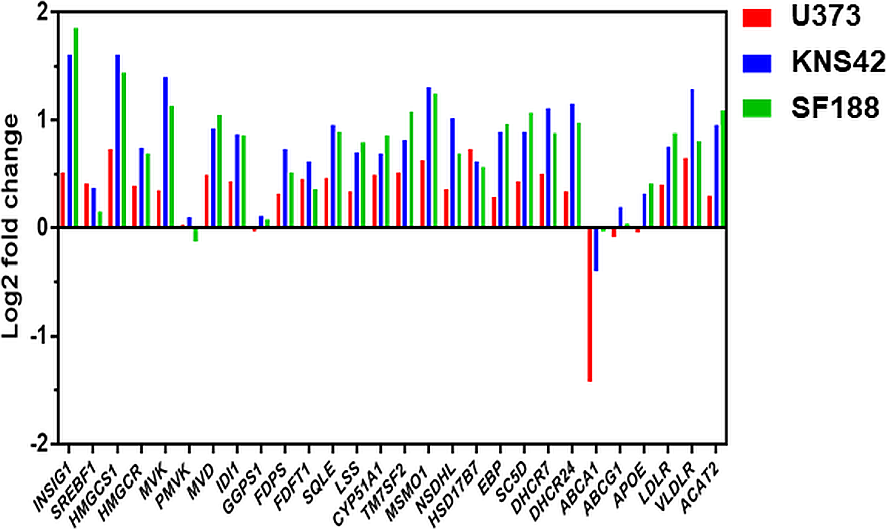

Supplement: Supplementary file 1 [file cancers-14-03873-s001.zip › Figure S2.tif]

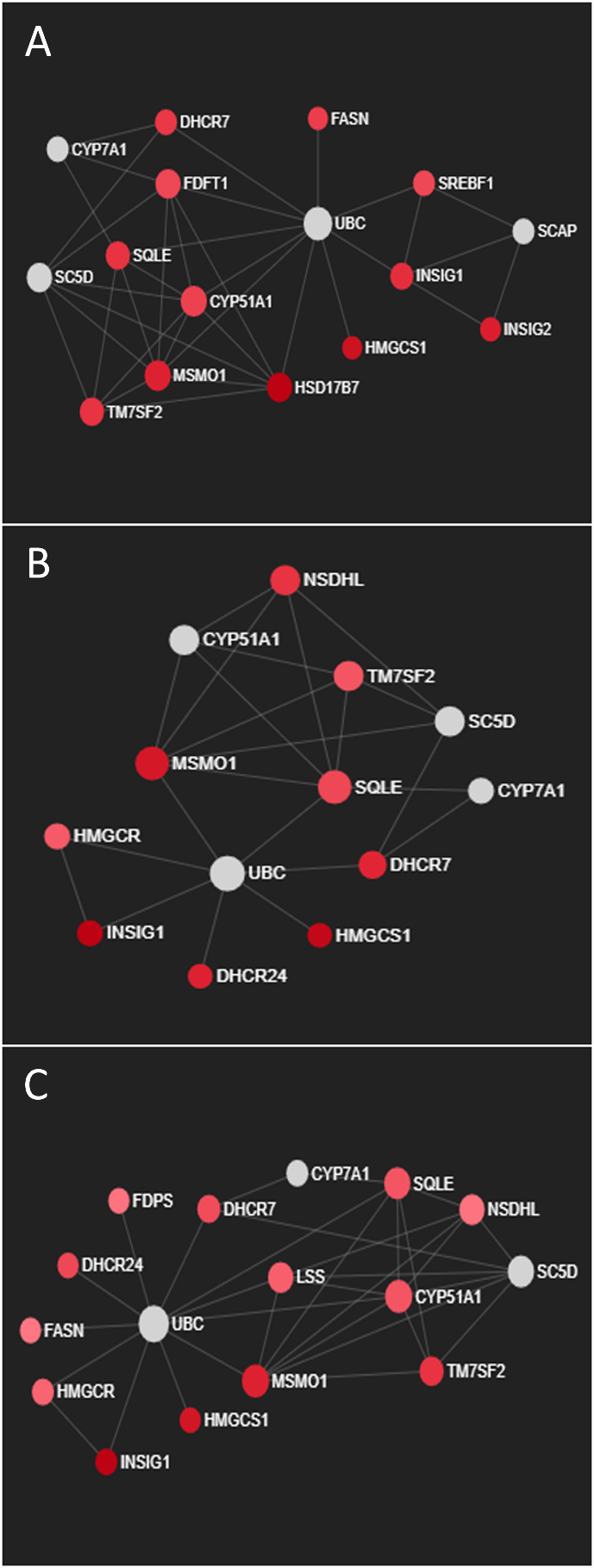

Supplement: Supplementary file 1 [file cancers-14-03873-s001.zip › Figure S3.tif]

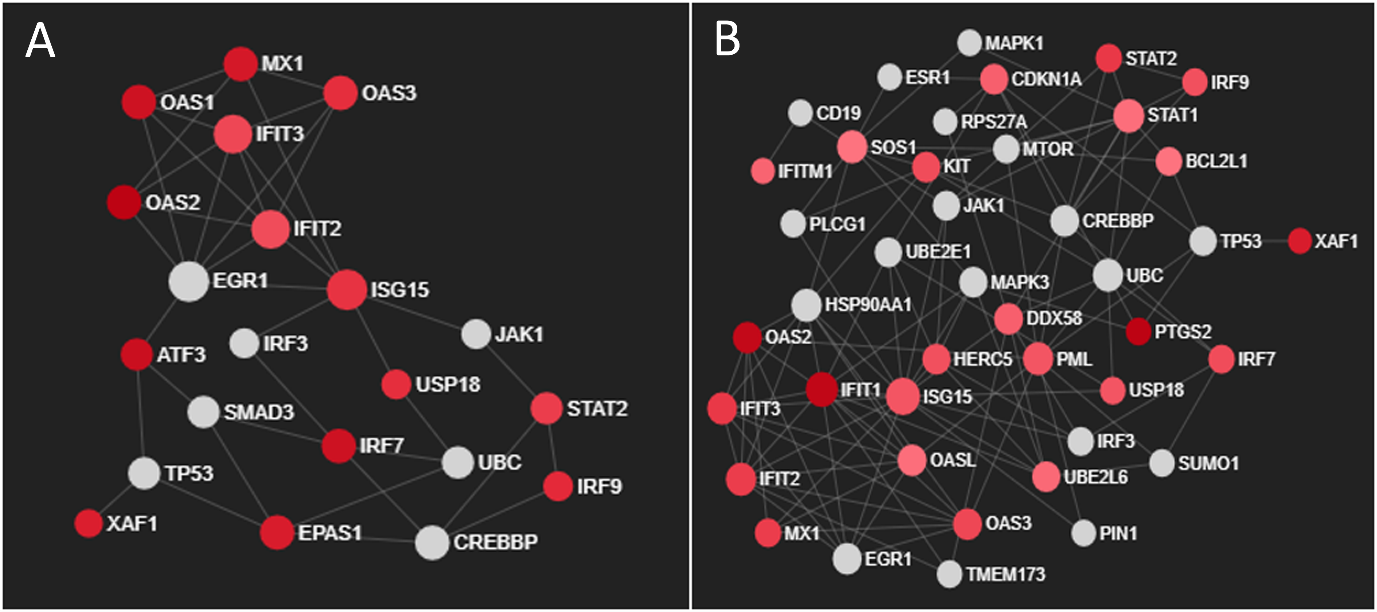

Supplement: Supplementary file 1 [file cancers-14-03873-s001.zip › Figure S4.tif]

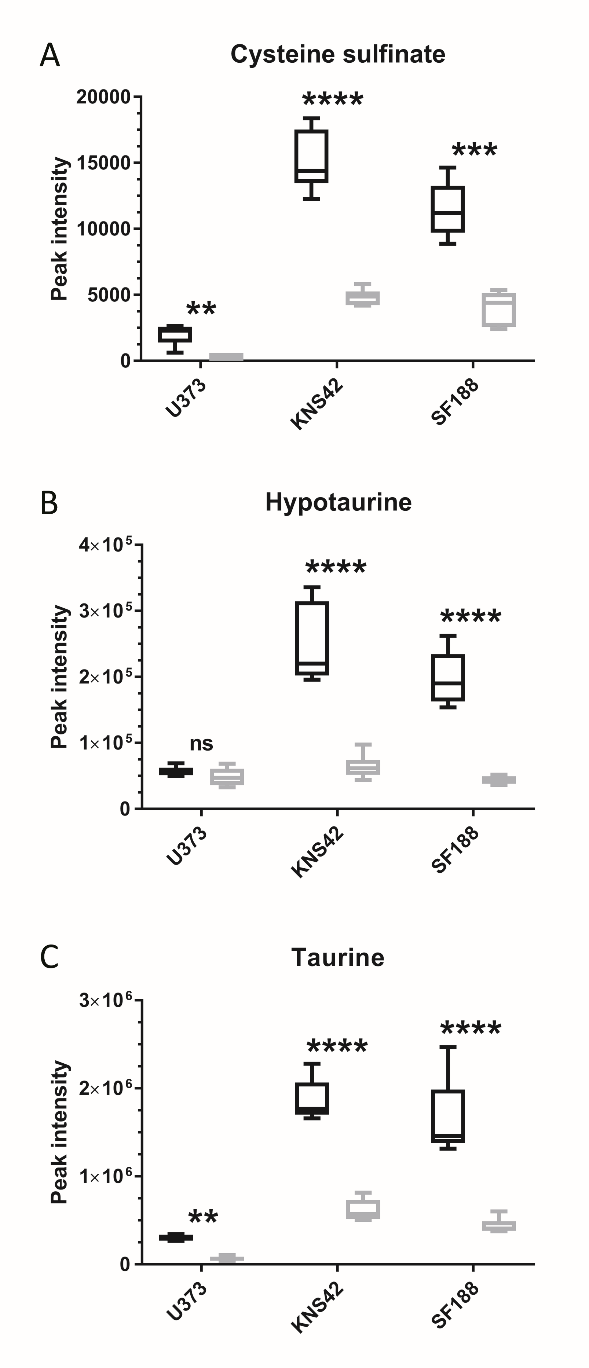

Supplement: Supplementary file 1 [file cancers-14-03873-s001.zip › Figure S5.tif]

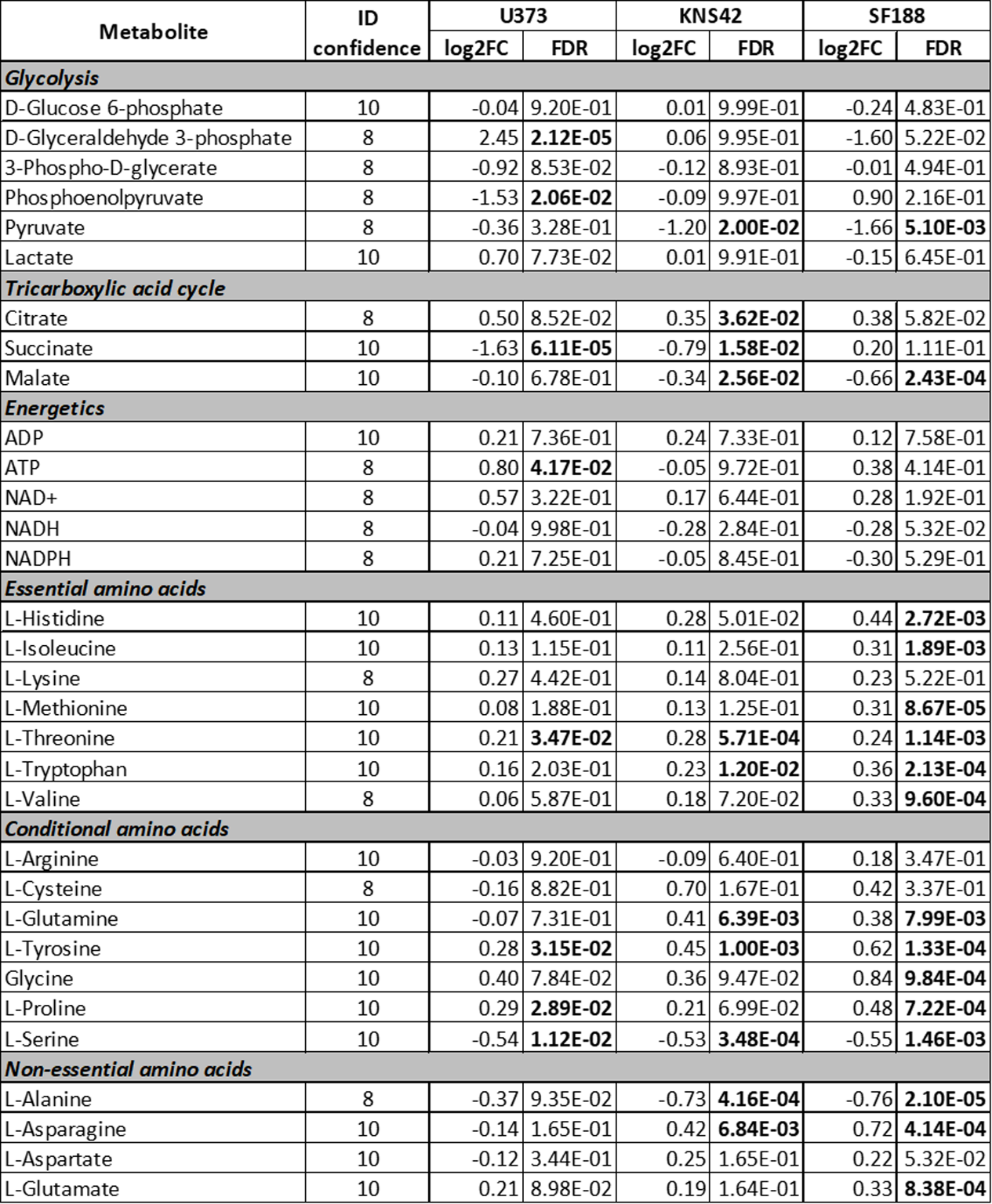

Supplement: Supplementary file 1 [file cancers-14-03873-s001.zip › Table S1.tif]

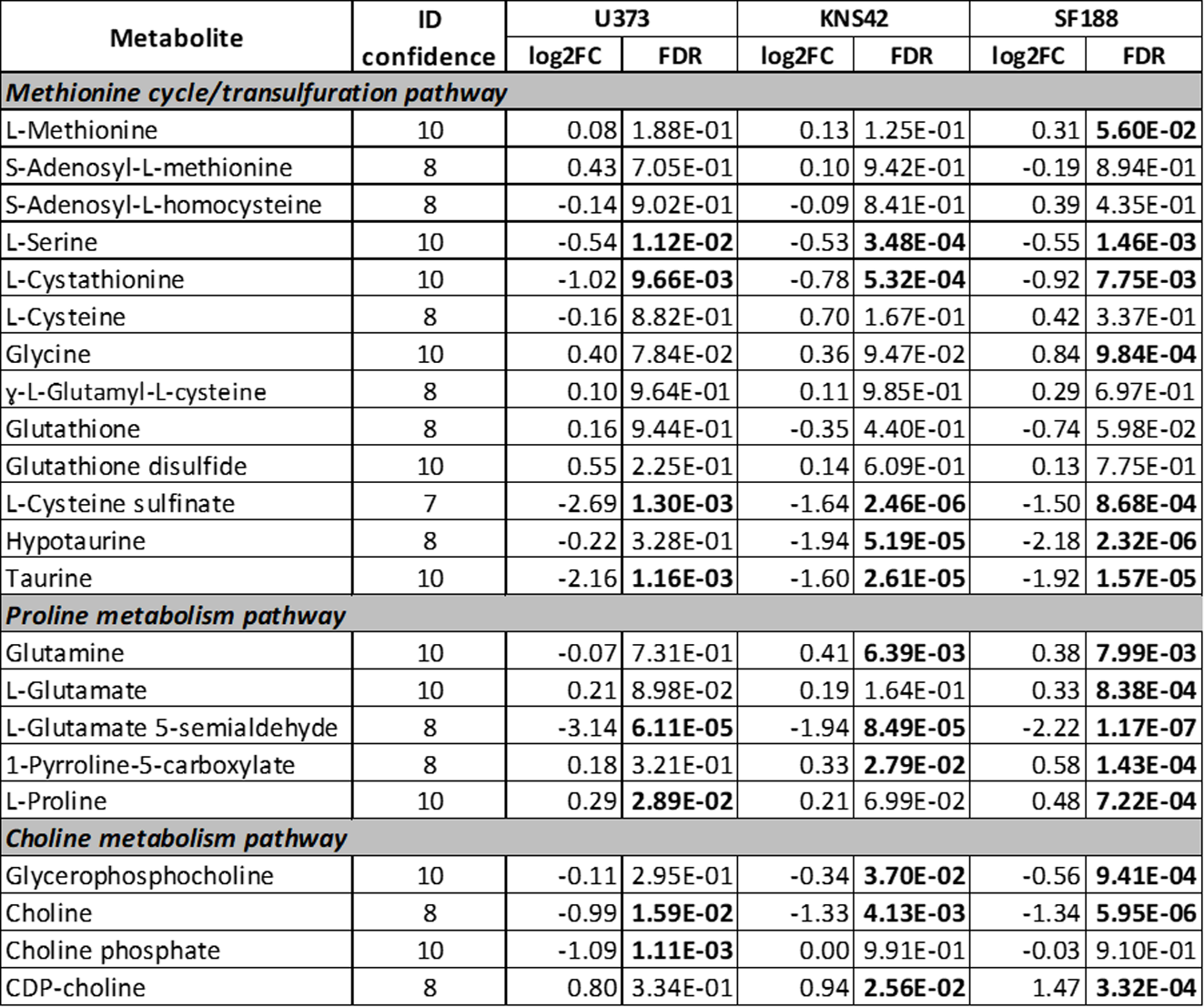

Supplement: Supplementary file 1 [file cancers-14-03873-s001.zip › Table S2.tif]

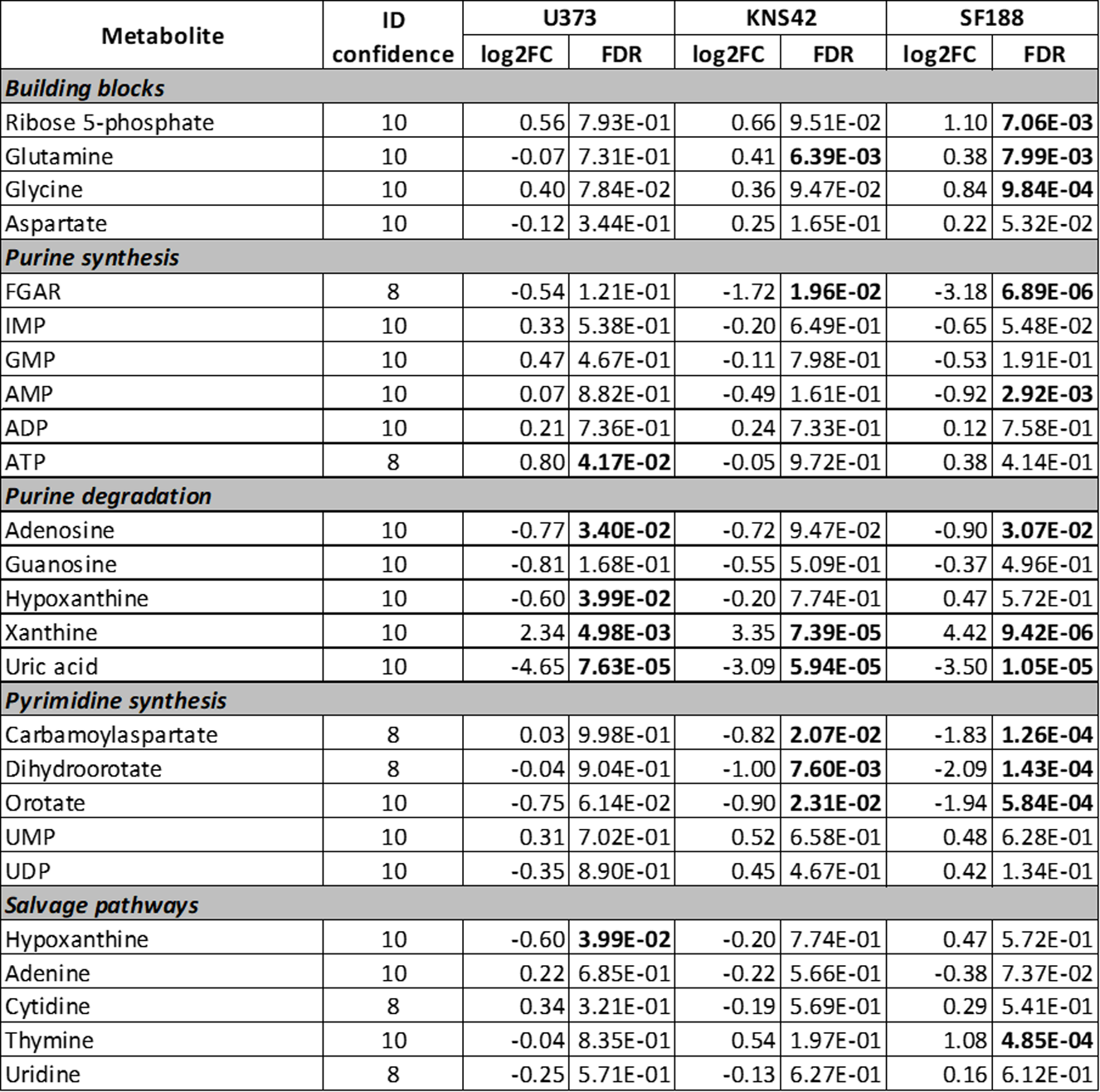

Supplement: Supplementary file 1 [file cancers-14-03873-s001.zip › Table S3.tif]

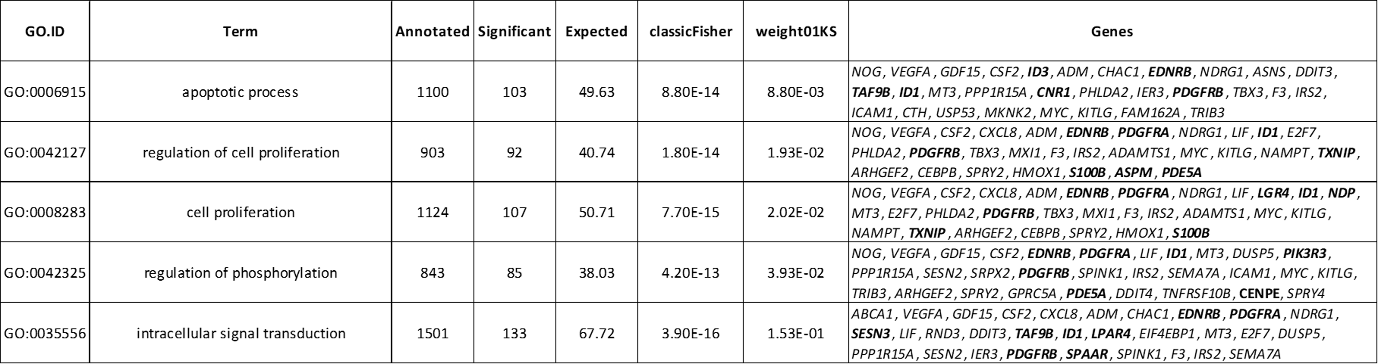

Supplement: Supplementary file 1 [file cancers-14-03873-s001.zip › Table S4.tif]

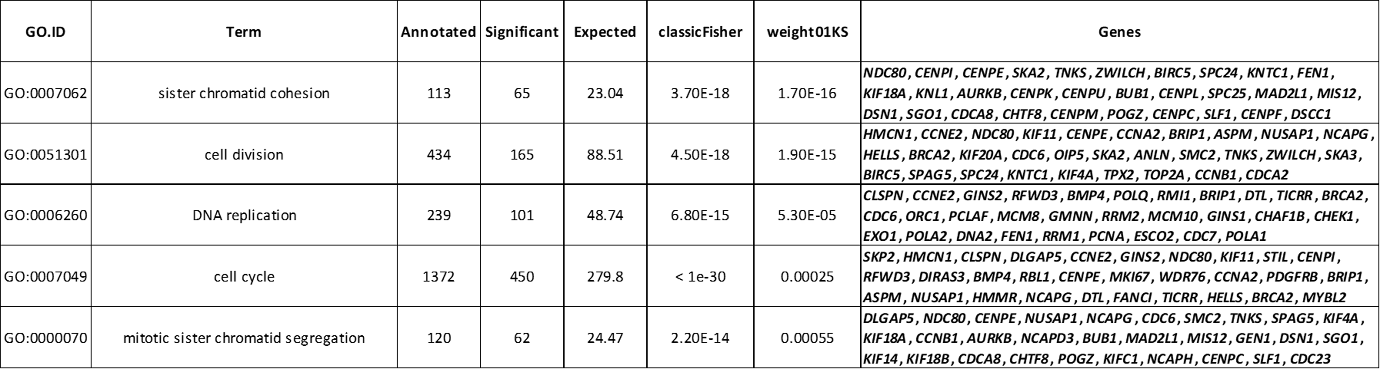

Supplement: Supplementary file 1 [file cancers-14-03873-s001.zip › Table S5.tif]

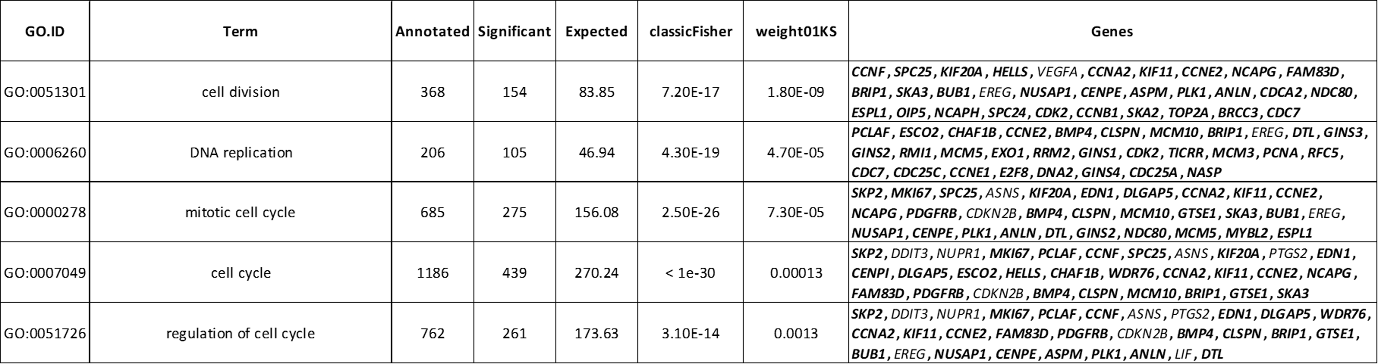

Supplement: Supplementary file 1 [file cancers-14-03873-s001.zip › Table S6.tif]
